# Modern Medico-Psychology and Psychiatry

**Published:** 1894-08-25

**Authors:** T. S. Clouston

**Affiliations:** Lecturer on Mental Diseases, Edinburgh University, and Physician Superintendent Royal Asylum, Morningside


### Modern Medico-Psychology and Psychiatry.

By T. S. Clouston, M.D., Lecturer on Mental Diseases, Edinburgh University, and Physician Superintendent Royal Asylum, Morningside.

### X.—CONDITION OF MENTAL EXALTATION AND EXCITEMENT.

If a grown-up man in ordinary circumstances were to exhibit constantly the mental liveliness, the talkativeness, the restlessness, and the small amount of self-control of a healthy child of six, he would be reckoned to be in a condition of morbid mental exaltation—of mania in fact. There are some brains so constituted that whenever the temperature rises much above 100 deg., they exhibit the mental signs of mania or delirium. It is in brains of such unstable quality too that we see undue exaltation, delirium, or fury, sometimes leading to crime, from small quantities of alcohol, opium, or Indian hemp, that would not so excite an ordinarily constituted brain. Every sort of morbid heightening of the mental energising might be classed under the "Conditions of Mental Exaltation" of the psychiatrist, but we practically confine those conditions to describe technical mania. This differs from melancholia essentially in there being no mental pain present, no affective depression. There may not be an exalted emotional state of joy in all cases of mania, but there is no consciously sorrowful feeling. In some cases there is morbid suspicion, or anger, or destructive fury, but the typical case of mania has real exaltation of feeling with diminished self-control over feeling, exalted brain working and uncontrolled conduct. The patient talks continuously in an elated and extravagant way. He is "excited" in the popular sense. Intellectually he may or he may not have delusions, but his mental processes are conducted so rapidly that they are not to be depended on. The patient may be brilliant in the early part of his attack, but he is wanting in balance and common sense. He may be very happy and express great love for his friends, but his affection has in it the elements of impulse, and is often foundationless. His conduct is always unnatural and changed. There are very marked distinctions between conditions of morbid depression and exaltation. In depression the patients always know they are ill, that there is something wrong. Commonly, they exaggerate their condition. In conditions of exaltation, on the contrary, the patients think themselves quite well, often imagining and ostentatiously affirming that they are better than they have ever been in their lives before. The other marked distinction between the two conditions is that in many of the milder conditions of depression the patients' business power and his common sense have not disappeared, whereas in conditions of exaltation they are not in the least to be depended on in regard to the conduct of their own affairs, and their common sense is lessened or altogether gone. Men may and do kill themselves in early melancholia; they often ruin themselves in early mania. I have had personal experience of many men and women making a wreck of their affairs and reducing themselves and their families to ruin when their brains were passing through a period of morbid exaltation by foolish speculation and investment, and by erratic conduct causing them to lose their situations, but I have seldom known a commencing melancholic to do so. He certainly does sometimes resign his position from fancied unworthiness to hold it, or because he thinks he has not done his work well. One of the most striking facts in regard to cases of morbid elevation of brain is the way in which the moral sense is changed, and the moral conduct altered for the worse; with this the social conventionalities are often strangely laid aside. One never realises how large a space conventionalities fill in our ordinary lives, and how closely related to the moralities and the important social relations they are, till one sees a man whose life has been honourable, precise, religious, businesslike, and conformable to the ordinary social codes of his class in life, become dishonourable, immoral, unsystematic, regardless of religious duties, careless as to his affairs, eccentric in dress, irregular as to meals, neglectful of appointments, dirty and slovenly in habits, yet perhaps acute intellectually and ready with excuses for his changed ways. One realises in such a case the thin partitions that divide the soundly working brain cells from their morbidly elevated condition of mild mania. The world in general does not reckon this condition, when it only exists in a mild degree, as insanity, and will not realise that it means irresponsibility of conduct, and that it needs watchfulness, care, and treatment. How many fortunes have I seen squandered because there is not physiological knowledge enough among even the intelligent public, or not elasticity enough in the legal conceptions of insanity to understand and cope with such really dangerous insanities. They are the "reasoning insanity" of the French and the "moral insanity" of Prichard, both being examples of mild morbid mental elevation.

It is not only the man's mind that is affected in such cases; his body shares in the morbidness. If its functions are carefully studied and compared with his normal healthy state, it will be found that the sleep is lessened, the appetites for food and drink and tobacco and their assimilation changed, the body weight and temperature altered, the skin and its secretions abnormal; all the lower, the baser and the more "animal" desires, that through heredity, evolution, and education have been kept under, will be found to have gained the mastery. Though the man may appear to be more social, this is commonly through a morbid accentuation of his social instincts which he is unable to regulate. He suffers from a want of power to be normally altruistic. The social instincts in him are so selfish for the time that the fabric of society, that has been built up through untold generations, would at once break up were all men in the same condition. It is often found that in this state the man's whole "character" has undergone a complete reversal, the quiet man becoming talkative, the modest man ostentatious, the meek man aggressive, and the penurious man a spendthrift. To a stranger, and for a short time, a man in this condition may appear brilliant and agreeable, but at home and to his relatives he is intolerable. One very curious fact I have frequently observed. The memory undergoes such a real accentuation that the man may be able to repeat long passages from authors that he has quite forgotten. I knew a man who during each attack of simple mania that he had coulp repeat many of Shakespeare's plays and many of the Psalms of David, which he could not do when well. He used to tell me afterwards that when in these morbidly exalted conditions he would at night vividly imagine himself to be Shakespeare as he declaimed his plays, or King David as he repeated the Psalms, and that this gave him great pleasure. The imagination is certainly greatly increased. The brain pace is increased enormously, but the break power is much lessened. The control may not be gone, however, and for short periods the patient may be able to "pull himself up," and speak in a natural way. This constitutes one difficulty of control and treatment in the present state of the law.

